# Correcting Cherenkov light attenuation in tissue using spatial frequency domain imaging for quantitative surface dosimetry during whole breast radiation therapy

**DOI:** 10.1117/1.JBO.24.7.071609

**Published:** 2018-11-10

**Authors:** Rachael Hachadorian, Petr Bruza, Michael Jermyn, Amaan Mazhar, David Cuccia, Lesley Jarvis, David Gladstone, Brian Pogue

**Affiliations:** a Dartmouth College, Thayer School of Engineering, Hanover, New Hampshire, United States; b DoseOptics LLC, Lebanon, New Hampshire, United States; c Modulated Imaging Inc., Irvine, California, United States; d Dartmouth-Hitchcock Medical Center, Lebanon, New Hampshire, United States; e Norris Cotton Cancer Center, Lebanon, New Hampshire, United States

**Keywords:** Cherenkov, spatial frequency domain imaging, tissue optics, radiation therapy, imaging

## Abstract

Imaging Cherenkov emission during radiotherapy permits real-time visualization of external beam delivery on superficial tissue. This signal is linear with absorbed dose in homogeneous media, indicating potential for quantitative dosimetry. In humans, the inherent heterogeneity of tissue optical properties (primarily from blood and skin pigment) distorts the linearity between detected Cherenkov signal and absorbed dose. We examine the potential to correct for superficial vasculature using spatial frequency domain imaging (SFDI) to map tissue optical properties for large fields of view. In phantoms, applying intensity corrections to simulate blood vessels improves Cherenkov image (CI) negative contrast by 24% for a vessel 1.9-mm-in diameter. In human trials, SFDI and CI are acquired for women undergoing whole breast radiotherapy. Applied corrections reduce heterogeneity due to vasculature within the sampling limits of the SFDI from a 22% difference as compared to the treatment plan, down to 6% in one region and from 14% down to 4% in another region. The optimal use for this combined imaging system approach is to correct for small heterogeneities such as superficial blood vessels or for interpatient variations in blood/melanin content such that the corrected CI more closely represents the surface dose delivered.

## 1 Introduction

Cherenkov emission exists across a spectrum of optical light emitted in low intensities throughout a given fraction of radiation therapy as a product of primary and secondary charged particles liberated during high-energy (MV range) x-ray or electron external beam therapy.^1^ Real-time verification of the treatment field area has previously been established in water tanks for quality assurance purposes, and verification with gamma index indicated excellent agreement with delivered dose.^2^ However, when imaging human tissue, the heterogeneity of patient absorbing and scattering features introduces nonlinearity between absorbed dose and Cherenkov emission signal.^3^ Monte Carlo simulations estimate that tissue absorption and scattering events can contribute up to 45% variation in the detected light.^3^ Therefore, the goal of this study is to determine whether Cherenkov-attenuating features may be corrected by accurate mapping of the tissue optical interaction coefficients gathered using a quantitative spatial frequency domain imaging (SFDI) system.^4^

During external beam radiotherapy, Cherenkov emission electromagnetically originates from secondary electrons, ionized with transferred speeds faster than the phase velocity of light in water and tissue, which are dielectric media. Cherenkov photons are generated in these media when the local electromagnetic field re-equilibrates after being disrupted by the electric polarization of relativistic Compton-scattered electrons. A Cherenkov emission spectrum in pure water spans from UV to NIR range (300 to 1500 nm), following an inverse square relationship $I\propto \frac{1}{\lambda^{2}}$, where I is the intensity and λ is the wavelength). On the other hand, the Cherenkov light emitted from tissue is detected predominantly in the 620- to 850-nm wavelength range, due to both high absorption of photons in the UV-green spectral range from natural tissue absorbers and low camera sensitivity in the infrared end of the spectrum.^5^ Prior to detection, the red and NIR Cherenkov photons are highly scattered and absorbed by blood within the vessels and melanin in the skin, disrupting the linearity between observed Cherenkov emission and deposited dose.^6^ Patient tissue optical properties vary globally from patient-to-patient, as well as locally, within each tissue. Spatial variation of detected Cherenkov light due to blood vessels is apparent and readily observed in the images.^3^ Additionally, temporally dependent changes, such as erythema from radiation burn side effects, will develop with varying degrees of severity in up to 85% of external beam radiotherapy patients.^7^^,^^8^ While other parameters contribute to image nonlinearity with dose, including surface curvature, field size, beam geometry, and source to surface distance, tissue optical properties contribute the most dominant variations (up to 45%) of all patient-specific parameters.^3^ Therefore, the goal in this study is to evaluate possible correction methodologies to compensate for tissue optical property variation. Addressing and correcting for the inconsistencies in Cherenkov imaging induced by tissue optical property variations could transform Cherenkov imaging from relative beam shape imaging to a quantitative dose delivery imaging system. The primary goal of this study was to determine if correction for subcutaneous vasculature and interstitial blood or pigment in the skin could improve the linearity between absorbed dose and the Cherenkov image (CI) intensity.

A recent development in quantitative tissue optical property imaging heavily oriented this study toward utilization of SFDI. Commercially available systems can provide both absorption and reduced scattering coefficients at each pixel over a wide field of view. The system used in our study employs a spatial light modulator to produce sinusoidal fringe patterns over a range of spatial frequencies across the surface of the skin, at three phase shifts for each frequency. Through model-based fitting, the optical property parameters absorption $\mu_{a}$ and reduced scattering $\mu_{s}^{\prime }$ are determined for each pixel.^9^ In turn, a calibration between effective attenuation and measured Cherenkov intensity can be established to enable correction of CIs over the absorption and scatter magnitudes exhibited by the patient’s tissue.

This is the first study to explore the compatibility of Cherenkov and SFDI images in tandem. Specifically, we establish a correction methodology, generate a calibration curve, validate the correction method on tissue phantoms, and finally demonstrate the correction of clinical CIs of a patient undergoing external beam radiotherapy. The long-term goal of this study is to correct for all variations in absorbing features, tissue surface curvature, and variations in Cherenkov emission due to differences in entrance versus exit beam dictated by preferential scatter at every pixel across the image. As a result, consistent Cherenkov emission intensity per cGy of absorbed dose would become independent of patient-specific tissue optical properties.

## 2 Materials and Methods

**Fig. 1:**
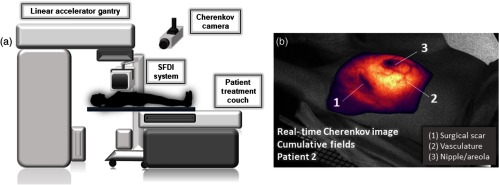
(a) A schematic of the *in vivo* setup shows the Cherenkov camera mounted to the ceiling and the SFDI system. (b) A cumulative CI acquisition is overlaid on a grayscale background image of Patient 2 as it appears in real time as the second of the two beams is delivered.

Figure 1(a) shows the clinical experimental setup, including the Cherenkov camera, linear accelerator, and the SFDI system. To note, the camera in the utilized treatment room is mounted to the right side of the patient and that the SFD imaging system is removed from the proximity of the accelerator as beam is delivered to the patient or experimental phantoms. Prior to patient acquisitions, the SFDI system perspective and field were aligned to match those of the Cherenkov camera. Rotational degrees of freedom were then marked on the system, and translational degrees of freedom were marked on the floor of the treatment room. At each clinical use, it was realigned using these markings to ensure translational accuracy. Figure 1(b) shows the cumulative CI as it is delivered in real time, superimposed on a background image of Patient 2. The most prominently attenuating features (nipple, vasculature, scar, etc.) are indicated.

### 2.1 Cherenkov Imaging

An intensified, gated, complementary metal oxide semiconductor (iCMOS) camera (C-Dose, DoseOptics LLC, Lebanon, New Hampshire) equipped with an AF Nikkon 50 mm f/2.8 lens (Nikon Inc., New York) was mounted on the ceiling at IEC 61217 spatial coordinates of (−1288, 1066, and −687) mm from the linac isocenter. The camera captured Cherenkov emission from each homogeneous phantom throughout 200 MU of a 6 MV external beam delivery, or each patient throughout their respective treatment plan. Gated operation of the image intensifier, which was enabled only throughout the duration of 4-μs x-ray pulses of the accelerator, allowed rejection of the room light signal in between each pulse. Each frame had a fixed exposure time of 51 ms, containing up to 18 linac pulses (in-sync frame), and was immediately followed by acquisition of an out-of-sync frame with 8 ms exposure time. During acquisition of the background, or out-of-sync frame, the intensifier was enabled over 720 ms with a 100-ms delay after the linac pulse, ensuring that only room light is recorded. Interleaved in-sync and out-of-sync frame acquisition enabled real-time x-ray noise filtering and room-light background subtraction, producing a clean Cherenkov emission video overlaid on live background video [Fig. 1(b)]. For each delivery fraction, the background-subtracted images were temporally summed across the duration of beam delivery, producing a cumulative CI that is independent of linac dose-rate fluctuations.

### 2.2 Spatial Frequency Domain Imaging

**Fig. 2:**
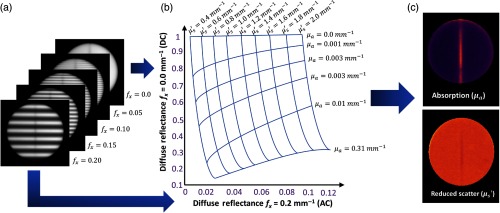
(a) One planar image and each of four spatial frequency images are taken at phases 0 deg, 120 deg, and 240 deg, per wavelength. This process is repeated for four wavelengths, where modulated images from only one phase and one wavelength is shown. (b) The LUT takes diffuse reflectance measurements and calibrates each reading based on an included phantom with known optical and reflectance properties. Reflectance is calibrated from both the planar frequency $f_{x}=0\;{\mathrm{mm}}^{-1}$, and one other spatial frequency, this pixel is matched with a specific absorption and reduced scatter pair using an LUT much like that pictured. (c) Optical properties are fit for each pixel (520×696), forming the images pictured.^10^

An SFDI system (Reflect RS, Modulated Imaging Inc., Irvine, California) was used to recover the optical properties of phantoms and patient tissue. SFDI fringe patterns with varying spatial frequencies (0.0, 0.05, 0.1, 0.15, and $0.2\;{\mathrm{mm}}^{-1}$) over three phases (0 deg, 120 deg, and 240 deg) were projected onto the target and imaged at 659, 691, 731, and 851 nm wavelengths. This system was customized to operate at a working distance of 42 cm and has field of view of 29  cm×22  cm. A sample image set of each of five spatial frequencies (one phase, one wavelength) is depicted in Fig. 2(a). The image was then acquired, and reflectance data were calibrated using a phantom of known optical and reflectance properties, yielding a calibrated reflectance $R_{d}$ for each wavelength at each pixel. The calibrated reflectance also includes a correction of surface topography based on previously published algorithms.^11^ The calibrated reflectance value is evaluated by the internal reflect RS homogenous Monte Carlo-based multiple-frequency lookup table (LUT)-based fitting algorithm to return corresponding $\mu_{a}$ and $\mu_{s}^{\prime }$ [Fig. 2(b)].^12^ The best fit will return a combination of $\mu_{a}$ and $\mu_{s}^{\prime }$. The $\mu_{a}$ and $\mu_{s}^{\prime }$ maps, such as those in Fig. 2(c), were generated with 520×696  pixel resolution.

Beginning with homogeneous phantoms, the SFD image was taken normal to the phantom surface to ensure focus across the entire surface of the phantom and to minimize artifacts due to phase unwrapping in height correction process. Each image took ∼30  s to acquire, followed by several minutes to process for optical property maps. The SFDI system was always removed from close proximity of the linear accelerator prior beam delivery. All Cherenkov and SFD image postprocessing and analysis were done in a MATLAB environment (Mathworks, Natick, Massachusetts).

### 2.3 Tissue Phantoms

Tissue-equivalent phantoms were used prior to patient imaging to confirm the range of intensities expected in both CIs and SFDI images and to generate the tissue optical property calibration curve.^13^ The phantoms consisted of water, Intralipid (soybean and egg yolk fat emulsion, Baxter Healthcare Corporation; Deerfield, Illinois), and blood (bovine whole blood in Na Heparin, Lampire Biological Laboratories; Pipersville, Pennsylvania), where blood concentrations were varied from 5% to 23%, and Intralipid remained fixed at 1% (diluted from 20%) and added at each iteration to compensate for increasing volume due to added blood. This equated to an effective attenuation coefficient range between 0.2 and $0.45\;{\mathrm{mm}}^{-1}$. The blood/Intralipid suspension was contained in a 5-cm diameter, 1-cm deep plate. A stir plate and pellet were used to ensure medium homogeneity for nonvessel tissue phantoms. The phantom container was painted matte black to reduce glare artifacts that arise during both Cherenkov and SFDI imaging.

In a second study, we validated and assessed the limits of our technique applied to an optically heterogeneous target using a range of synthetic vessel phantoms. The blood vessels were simulated using clear polytetrafluorethylene (PTFE) tubing with 100% blood, diameters of 0.8, 1.9, and 2.3 mm and respective wall thicknesses of 0.46, 0.3, and 0.3 mm. The tubing was fixed in a black-coated well plate with a diameter of 14 cm and secured with a sealing glue. The surrounding media was constituted by a 0.5% blood and 1% Intralipid suspension to mimic properties of adipose tissue.^10^ These phantoms contained an adjacent length of tubing parallel to the tubing containing blood, ∼2  cm to the left, filled with bulk media. These measures were carried out to ensure that the amount of Cherenkov light coming from the tubing did not compromise the integrity of the signal from the sample. This was the case for the first selection of tubing, which led to the selection of thinner (PTFE) tubing that incited far fewer (if not negligible) Cherenkov photons upon irradiation. The well plate provided a sufficient volume of media beneath the tubing to incite underlying Cherenkov emission from 5 mm below the vessel, and the starting measurement was taken with enough media to cover the surface of the tubing. An SFD image was taken for this phantom, then ∼15.2  mL of media was added to superimpose 1 mm of media on top of the vessel, further submerging it. This process continued every 1 mm until the vessel was submerged beneath 5 mm of media. Manual mixing at each step ensured homogeneity of the phantom surrounding the vessel. After completing SFD imaging, the superimposed media was removed and this process was repeated for the same series of Cherenkov acquisitions, ensuring that the placement of the imaging system and the sample remained the same between additions of media, facilitating a robust image registration.

### 2.4 Patient Radiation Therapy

All human imaging was done under an approved Institutional Review Board (IRB) protocol at Dartmouth College, and all procedures were carried out as described in this protocol. The recruitment to participate included informed consent for imaging the treatment area before and during radiotherapy. All subjects were treated by their clinically prescribed whole breast radiotherapy plan using beams from angles optimized to minimize damage to healthy tissue. These include right posterior (RP), left anterior (LA), right posterior oblique (RPO), and left anterior oblique (LAO) beam angles, prior to or following breast conserving surgery. Patient 1 was prescribed a hyperfractionated plan, 5 fraction/week treatment over 6 weeks, 4 beams centered around the breast, LAO (47 cGy), LA (50 cGy), RPO (42 cGy), RP (41 cGy), and two superclavicular fields, RPO (152 cGy), and LAO (28 cGy). Patient 2 was prescribed a standard hypofractionated plan, 5 fraction/week treatment over 4 weeks, which included a delivery of two beam angles, RPO (137 cGy) and LAO (129 cGy) at each fraction by a Varian CD2100 Linac (Varian Medical Systems Inc., Palo Alto, California), incorporating both 6 and 10 MV beams in most patients. All treatments were carried out at the Norris Cotton Cancer Center, a component of Dartmouth-Hitchcock Medical Center (Lebanon, New Hampshire).

### 2.5 Theory

The empirical foundations upon which this study is based rely on, namely, the validity of diffusion theory in homogeneous media and Beer’s Law. We construct a tissue optical property correction technique in the general form of $I_{0}=I/\mathrm{CF}(\mu_{\mathrm{eff}})\;$, where I is the detected or observed Cherenkov light intensity, $I_{0}$ is the corrected light intensity, and $\mathrm{CF}(\mu_{\mathrm{eff}})$ is a correction function, dependent on the effective attenuation coefficient. It is logical to imply that this correction function must also depend on tissue heterogeneity and on Cherenkov source distribution. For clarity and due to the limitations of our imaging techniques, we avoid resolving the Cherenkov source depth distribution by introducing an average sampling depth d and assume that the tissue is optically homogeneous in depth.

To facilitate clinical tissue optical property correction, a calibration curve was constructed from the relationship between detected Cherenkov emission and measured effective attenuation. From the reduced scatter and absorption maps provided by the SFDI system, the effective attenuation coefficient maps were generated, given the approximation in diffusion theory

$$u_{\mathrm{eff}}=\sqrt{3\mu_{a}\mu_{s}^{\prime }},$$ (1)

characterized by the absorption ($\mu_{a}$) and reduced scatter ($\mu_{s}^{\prime }$) coefficients. This expression in diffusion theory is valid as long as $\mu_{a}\ll \mu_{s}^{\prime }\;$ within the tissue/phantom. Because light-exciting tissue is largely governed by this attenuation, it was hypothesized that a single correction factor could be applied to each pixel within the field of view imaged to correct for tissue optical properties.

The exponential decay relationship in consideration is illustrated by the Beer–Lambert law:

$$C=C_{0}\cdot \exp[-\mu_{\mathrm{eff}}\cdot \left\langle d\right\rangle ],$$ (2)

where C represents detected Cherenkov light, $C_{0}$ is the initial Cherenkov light, and ⟨d⟩ is the mean sampling depth of Cherenkov emission. The correction of each Cherenkov count value using the discussed methodology is straight-forward: to invert the exponential and solve for $C_{0}$ from measured C and $\mu_{\mathrm{eff}}$,

$$C_{0}=C\cdot \exp[\mu_{\mathrm{eff}}\cdot \langle d\rangle ],$$ (3)

where d can be estimated from fitting the tissue phantom data. The correction proposed in Eq. (3) then removes the attenuation at each pixel. In linear form, this becomes

$$C_{0}=\exp[\ln(C)+\mu_{\mathrm{eff}}\cdot \left\langle d\right\rangle ].$$ (4)

For each pair of corrected and uncorrected images, the contrast was computed by taking the mean intensity value of an ROI inside the vessel over the mean intensity of an ROI of the surrounding bulk media. This metric is used to ensure consistency across calculations by normalizing to the conditions of the surrounding media environment. The same procedure is carried out for *in vivo* vessels in patient images with an additional step, which includes comparison to the treatment plan, such that the expected dose gradient of the plan is considered. Finally, all images are coregistered to ensure the same ROI is being evaluated.

## 3 Results

### 3.1 Homogeneous Phantom Calibration Curve

**Fig. 3:**
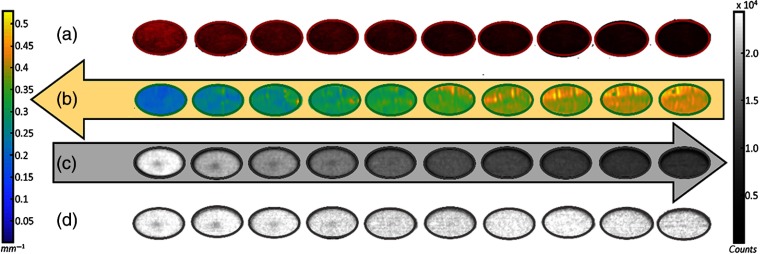
(a) An observable increase in effective attenuation follows a series of 5% to 24% blood concentrations (first row) where blood is a known optical light absorber. (b) SFDI absorption and scatter maps were taken for each sample and used to create effective attenuation maps using the expression in Eq. (1). (c) The respective CIs illustrate the reduction in Cherenkov light with increasing $\mu_{\mathrm{eff}}$. (d) Once the correction has been applied, the average value of each well equalizes to 3.81×104 counts with a standard deviation of 879 counts, CV (σ/μ)=0.023. The decreased Cherenkov intensity in the center of each well is an artifact due to the stir pellet.

**Fig. 4:**
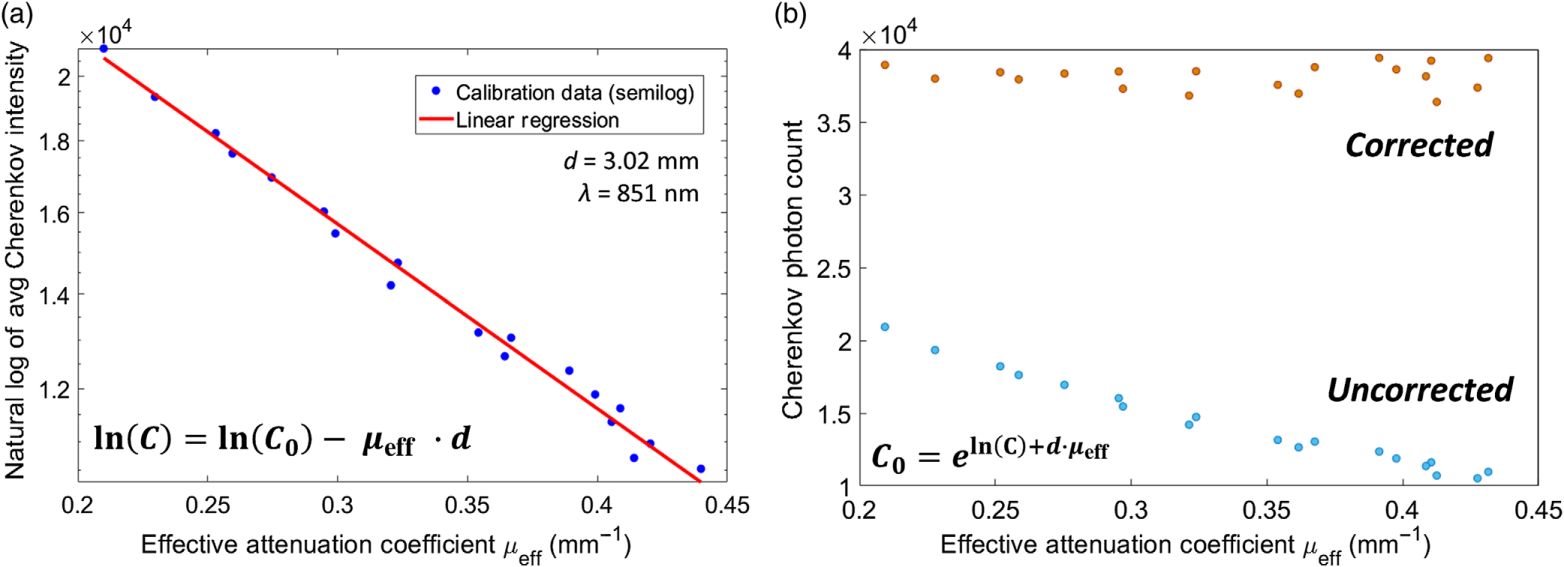
(a) The calibration data set between SDFI-measured effective attenuation coefficients (*in vivo* range, 851 nm) and the associated average detected Cherenkov outputs (natural log scale). The measured Cherenkov emission ln(C) and effective attenuation ($\mu_{\mathrm{eff}}$) pair are used to extrapolate the intercept $C_{0}$ or $C_{\text{corrected}}$, where $C_{\text{corrected}}$ and effective sampling depth *d* have been fit linearly. (b) The uncorrected average ROI values of each phantom (blue) illustrate exponential decay with increasing blood concentration or $\mu_{\mathrm{eff}}$ and compares to its corresponding value with the correction function applied (orange).

Highly absorbing features, such as blood and skin pigment, have a vast influence over observed Cherenkov intensity, illustrating reductions up to 60% in the calibration data set. In Fig. 3(a), a white light image of each homogeneous phantom is shown over the series of increasing blood concentrations (top row). Below, the respective effective attenuation map [Fig. 3(b)] and CI [Fig. 3(c)] is recovered for 851 nm central wavelength. As effective attenuation increases (indicated by color bar, left), the fluence of the Cherenkov emission decreases (color bar, right). The mean values of measured Cherenkov optical intensity and effective attenuation coefficients were used to construct the calibration curve in Fig. 4(a), by way of Eq. (4). This data set was fit using each of the four NIR wavelengths used by the SFD imaging system, where the calibration at 851 nm illustrated the best fit and was thus chosen for application. This confirmed an exponentially decreasing emission of Cherenkov light with an increasing blood concentration, and therefore, $\mu_{a}$ and $\mu_{\mathrm{eff}}$. To demonstrate this proof of this concept, the fit used to determine the blood–Intralipid phantom calibration is applied back to the same data set, to illustrate the extent of Cherenkov emission correction in an ideal case [Fig. 4(b)]. The corrected Cherenkov intensities approach values that would be measured in a phantom with virtually no absorption. In this particular case, we observed a mean corrected intensity of $\mu =3.8\times 10^{4}$ counts, with a standard deviation of σ=879 counts. The coefficient of variation (CV=  σ/μ) equaled ∼2.3%. (Note this μ as an average and is not to be mistaken for an attenuation coefficient.)

### 3.2 Verification of heterogeneous phantoms

**Fig. 5:**
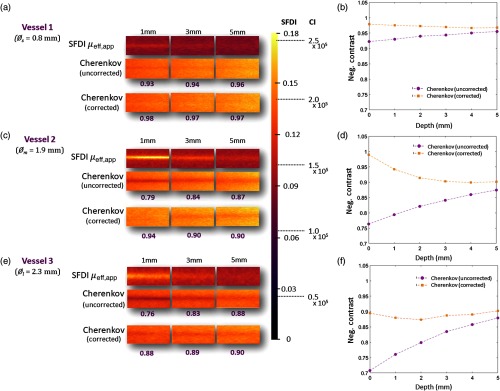
In (a) (small vessel case, ${Ø}_{s}=0.8\;\mathrm{mm}$), (c) (medium vessel case, ${Ø}_{m}=1.9\;\mathrm{mm}$), and (e) (large vessel case, ${Ø}_{l}=2.3\;\mathrm{mm}$), the SFD image of each vessel is organized at the top of the phantom’s respective CI both uncorrected (middle) and corrected (bottom). The uncorrected CI more clearly shows the synthetic vessel region through the center of each image, whereas the corrected image shows the diminishing of local differences between the two regions after having applied the pixel-by-pixel correction factor, dictated by Eq. (4). The associated negative contrast ($I_{\mathrm{vessel}}/I_{\mathrm{medium}}$) is listed under each CI. The color bar represents scaling for both modalities, where the SFDI axis and CI axis are shown to its right. In (b), the small vessel (negative contrast values are organized into two plots: corrected and uncorrected, as well as for the case for (d) a medium sized vessel ${Ø}_{m}=1.9\;\mathrm{mm}$ and (f) a large vessel ${Ø}_{l}=2.3\;\mathrm{mm}$.

Once the correction technique was verified, it was applied to a phantom series featuring synthetic blood vessel inclusions with varying inner diameters (${Ø}_{s}=0.8\;\mathrm{mm}$, ${Ø}_{m}=1.9\;\mathrm{mm}$, and ${Ø}_{l}=2.3\;\mathrm{mm}$), and contrasts were compared between corrected and uncorrected images. In Fig. 5(a), spatial frequency domain images recover the effective attenuation coefficient $\mu_{\mathrm{eff}}$ in the location of blood vessel (top row). The uncorrected CI (row 2) shows attenuated signal in the same region. The two images are registered, and the correction factor defined in the denominator of Eq. (4) is applied to the CI (third row) using the corresponding intensity values from the SFD image. With each iterative addition of superimposed media, the observable attenuation of the Cherenkov signal diminishes in response to increased signal from “tissue” overlying the attenuating vessel, and the SFDI $\mu_{\mathrm{eff}}$ signal attenuates as the primarily absorbing vessel becomes covered by highly scattering media. For the smallest vessel [${Ø}_{s}=0.8\;\mathrm{mm}$, Fig. 5(a)], the negative contrast increases from 0.92 to 0.98 in the case for 0 mm [Fig. 5(b)]. When 5-mm depth in media is reached, the negative contrast between the corrected and uncorrected CI differs by only 1%. In the next largest vessel [${Ø}_{m}=1.6\;\mathrm{mm}$, Fig. 5(c)], negative contrast increases from 0.76 in the uncorrected image to 0.99 in the corrected image at 0 mm, illustrating the best correction and most dramatic improvement [Fig. 5(d)]. At 5 mm, the negative contrast decreases to a 3% difference between corrected and uncorrected images. In the case of the largest vessel [Fig. 5(e)], the minimum negative contrast achieved for the corrected vessel is 0.9 in both cases where vessel depth = 0 and 5 mm. The negative contrast percent difference increases from 29% at 0 mm, to 2% at 5 mm [Fig. 5(f)].

### 3.3 Verification of Patient Images

**Fig. 6:**
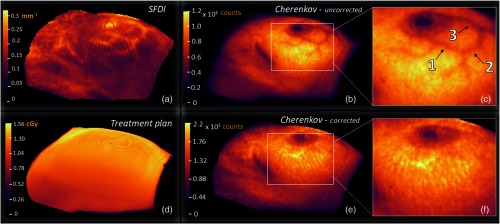
In (a), the effective attenuation optical property map is generated and masked to include pixels at 20% the maximum Cherenkov value or greater, as shown in (b). The uncorrected CI exhibits darker regions due to near-surface vasculature, the nipple, and surgical scar. The attenuation introduced by blood absorption due to subcutaneous blood vessels yields a contrast percent difference of up to 19%, emphasized in (c). In (e), the corrected CI is adjusted, pixel-by-pixel, using the methodology discussed in Eq. (4), and magnified in (f) to again emphasize the region, where the SFDI is best in focus. To illustrate an ideal theoretical case, the 5-mm subsurface dose is read from DICOM format, aligned to the same view angle as of Cherenkov and SFD cameras, rendered in VTK, and exported from the Cherenkov imaging software (d).

The technique was then applied to data acquired from Patient 2. The SFDI effective attenuation map in Fig. 6(a) is masked to include only pixels within a height and angle tolerance acceptable for height correction algorithms. In Fig. 6(b), the uncorrected CI shows the presence of vasculature due to attenuation of Cherenkov light in the hemoglobin content of the blood. The other two most prominent features include the nipple and the surgical scar, both also highly attenuating due to variations in pigment content. In Fig. 6(e), the correction of attenuation due to vasculature is showcased, then magnified in Fig. 6(f). As shown in Figs. 6(c) and 6(f), the vasculature in this region of the CI from a fraction of Patient 2’s treatment appears to be completely corrected for, qualitatively. Further, Fig. 6(d) shows the rendered dose map (VTK) associated with one fraction of treatment from the perspective of the Cherenkov camera, sampled down to ∼5  mm, weighted by a short buildup region, followed by exponential falloff.

An ROI mean was taken at three vessel locations inferior to the nipple [indicated in Fig. 6(c)] and at regions directly adjacent to those in order to determine negative contrast values (Table 1). Because negative contrast does not describe a metric of success, a percent difference was taken between values in corrected and uncorrected images and compared to the patient treatment plan, such that the slight intensity gradient associated with changes in dose deposition between ROIs would be accounted for. It becomes evident from Table 1 that percent differences associated with the corrected image lateral, inferior, and medial vessels (6%, 4%, and 7%, respectively) adhered more closely to the intensity gradient associated with the treatment plan, compared to that of the uncorrected image (22%, 14%, and 10%, respectively). Although the medial case does quantify an improvement, the change is notably less than those illustrated by ROI 1 and 2.

**Table 1:** The CI contrast between a small ROI inside a region attenuated by the vessel and a nearby region adjacent to the vessel are organized into rows 2 and 3, separated by the corrected and uncorrected values (bold). The first row organizes contrasts associated with the same regions of the coregistered treatment. Percent differences between contrasts for each CI ROI and respective treatment plan ROI are provided in the appropriate box.

	Lateral vessel (ROI 1)	Inferior vessel (ROI 2)	Medial vessel (ROI 3)
Treatment plan	**1.01**	**1.00**	**0.98**
Cherenkov (uncorrected)	**0.79**	**0.86**	**0.88**
	${\Delta \mathrm{NC}}_{\mathrm{tp}}=-22\%$	${\Delta \mathrm{NC}}_{\mathrm{tp}}=-14\%$	${\Delta \mathrm{NC}}_{\mathrm{tp}}=-10\%$
Cherenkov (corrected)	**0.95**	**0.96**	**0.91**
	$\Delta {\mathrm{NC}}_{\mathrm{tp}}=-6\%$	${\Delta \mathrm{NC}}_{\mathrm{tp}}=-4\%$	${\Delta \mathrm{NC}}_{\mathrm{tp}}=-7\%$

### 3.4 Dynamic Nature of Tissue Optical Properties

**Fig. 7:**
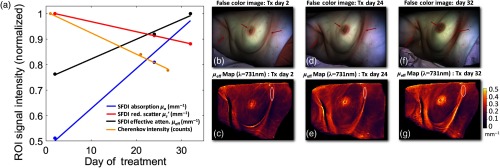
(a) The breakdown of changing tissue optical properties in Patient 1, which are normalized and plotted over the course of one month of treatment (beginning, middle, and end), where SFDI data are taken at days 2, 24, and 32, and Cherenkov at days 1, 21, and 27. The region analyzed is indicated by a red circle in the mammary fold in the bottom row of effective attenuation maps (c), (e), and (g). Absorption in this region increases by 49%, and reduced scatter decreases by 13%, yielding an overall effective attenuation change of ∼24%. Measured Cherenkov emission, conversely, decreases by ∼22%. Although these changes are not illustrated in full-color format, the false-color images reconstructed using the NIR wavelengths employed by the reflect RS clearly indicate the changes incited around the mammary fold, as well as those in the axillary fold and the nipple-areola complex (b), (d), and (f).

SFDI measurements show that tissue optical properties varied significantly throughout the course of a hyper-fractionated radiotherapy treatment for Patient 1. In Figs. 7(b), 7(d), and 7(f), the axillary and mammary fold regions illustrate where erythema due to irradiation is most evident. The associated color images were reconstructed into RGB channels using the modulated images at each phase and spatial frequency of the wavelengths employed using the NIR setting of the SFDI system (649, 691, 731, and 851 nm) and the demodulation equation. ^14^ Because blue wavelengths are neglected, the product is essentially a “false color” image. Figure 7(a) shows the tissue optical property breakdown sampled from the ROIs delineated in white from the mammary fold just inferior to the breast [(c), (e), and (g)]. It was shown that reduced scatter decreased over the course of the treatment by ∼13%, and absorption increased by ∼49% for Patient 1. A similar ROI evaluation of the measured Cherenkov light shows substantial Cherenkov falloff as treatment progresses. Overall, the effective attenuation increased by ∼24%, and the corresponding Cherenkov emission decreases by 22%. Going forward, it is important to acknowledge the highly dynamic and temporally dependent nature of tissue optical properties throughout ongoing radiation therapy.

## 4 Discussion

Figure 4(a) depicts that the relationship between SFDI-acquired, effective attenuation, and Cherenkov emission from a homogeneous phantom follows an exponentially decaying trend. After the Cherenkov intensity correction technique was applied to the mean intensities of each homogeneous phantom [Fig. 4(b)], the percent coefficient of variation was ∼2%.

Cherenkov emission for the best heterogeneous phantom case (Fig. 5) improved by ∼27% for the medium-sized vessel at 0 mm. The negative contrast values of the Cherenkov emission stayed within 12% of the ideal negative contrast of 1. It should be understood that our 0-mm depth is used to establish a baseline, where media was added until enough to visibly cover the vessel was superimposed. This is to mimic the ∼0.1-mm thickness of the epidermis, which contains no vasculature, and thus a feasible start point. The SFDI system’s depth sampling limit is ∼4  mm depth, as documented in the manufacturer’s manual. In our case, the effective sampling depth parameter d, where from Fig. 4(a),d=3.02  mm. This factor is also dependent on the spatial frequencies evaluated.

This serves as an explanation as to why the negative contrast associated with the largest diameter vessel remained farthest from 1. With no media superimposed, it begins with the distal edge of the tube already near the sampling limit while attenuation due to Cherenkov is still observable at 5 mm. In Figs. 5(d) and 5(f), this is shown as the difference between corrected and uncorrected negative contrasts begin to stabilize as lower depths.

Our vessel phantoms evaluated the feasibility of initially correcting for a thick vessel at the surface (where the highest magnitudes of Cherenkov attenuation would be observed and the highest $\mu_{\mathrm{eff}}$ signal) down to a small vessel, far from the surface (smaller magnitudes of Cherenkov attenuation and attenuated $\mu_{\mathrm{eff}}$ signal). The former recreated a more difficult correction scenario than encountered *in vivo*, and results illustrate that this attenuation is not flawlessly corrected for. Although SFDI and CI serve as compatible modalities for surface dose imaging, it is evident from this study that clinically relevant correction of Cherenkov emission (3% to 5% precision) for subcutaneous vasculature and bulk tissue optical properties to establish linearity between Cherenkov and absorbed dose is challenging using a single-wavelength SFDI information. This is most likely due to the compression of the spatially varying optical properties to a single value of $\mu_{\mathrm{eff}}$. In combination with such a spatially distributed source of Cherenkov emission, the depth insensitivity plays major role in the remaining 12% imprecision observed in this experiment. By employing a single NIR wavelength of the four available from the SFDI system (in contrast to the broad VIS-NIR Cherenkov spectrum reaching past 850 nm), there is a disproportionality associated with contributions of light as a function of depth. Although the calibration established at 851 nm most closely correlated the two modalities, the lost depth information may be responsible for the incomplete CI correction. In response to this finding, evaluating a spectrally weighted calibration is planned for future works.

Furthermore, surface curvature in the *in vivo* case (an inherent property of breast tissue and the surrounding skin) forces certain regions around the periphery of the SFDI image field to become out of focus. It is expected that this is partly behind a lesser correction in ROI 3 of Table 1 (medial vessel), where the difference between corrected image and treatment plan compared to uncorrected image and treatment plan negative contrasts was only 3%.

Another related consideration involves correcting for the periphery of the Cherenkov field, i.e., why in each CI, the intensity gradient [Figs. 6(b) and 6(e)] appears to fall off more dramatically than each surface dose gradient [Fig. 6(d)]. This study focuses on corrections within regions of the image that can be trusted to be in-focus and centrally located. This asserts that the curvature of the breast incites difficulty when evaluating linearity between the modalities. On the other hand, if we focus on the region most normal to the imaging plane [Figs. 6(e) and 6(f)], the CI attenuation due to the vasculature was completely corrected. Correcting the periphery of the field exists among the parameters that we will address in future works.

It is suggested further that lack of appropriate linearity between SFDI signal and corresponding Cherenkov attenuation in subcutaneous vessels could depend on variation between tissue composition of the patient breast, specifically, by density. The adipose content of fattier breasts may allow more Cherenkov light to escape from deeper within the tissue, thereby introducing a more dramatic negative contrast between vessel and surrounding media. Conversely, in denser breasts, the Cherenkov emission may be minimized, and therefore, the negative contrast between vessel and surrounding tissue. Both patients in this study were characterized as having scattered fibroglandular tissue, as evaluated from gadolinium-enhanced MRI scans. A deeper analysis of tissue density 5 to 7 mm below the skin surface its effect on emitted Cherenkov intensity is currently underway.

As previously discussed, registration of both CI and SFDI images to ensure vasculature and other feature alignment is critical for pixel-by-pixel correction. If misaligned, adjacent image pixels will be left under- and over-corrected. Therefore, this study recommends either robust image registration using mutual information or manual, point-by-point, fiducial selection.

The temporally variant optical property findings addressed in Fig. 7 were also of considerable importance, and therefore, should be addressed for robust CI corrections. Evidence of the most adverse effects on the skin by MV beam are readily discerned and increase in severity from day 2 through day 32, which illustrate that regardless of modality used to measure optical properties, they tend to change markedly over the course of a typical radiotherapy treatment. It is, therefore, implied that the tissue optical property measurement should be performed ideally during each Cherenkov imaging session.

Correcting entirely for subcutaneous vasculature and bulk tissue optical properties to establish linearity between Cherenkov and absorbed dose will not be possible using exclusively the methods applied in this paper. Instead, a more sophisticated layered model would be necessary. Whether or not differences in skin pigment can be aggressively corrected has yet to be determined, but evaluation is intended for later works.

## 5 Conclusions

Imaging Cherenkov emission during radiotherapy has been developed to establish real-time field verification *in vivo*. However, establishing real-time quantitative dosimetry remains a complex and dynamic problem involving many parameters. This study investigated the first step toward correcting for tissue-derived optical attenuation using wide-field SFDI registered to each cumulative CI and used to generate and apply pixel-by-pixel maps of corrective scale factors. In summary, this study has not only addressed the contributions and pitfalls of CI correction using SFDI but has also revealed several important considerations regarding the correction of CIs in the context of establishing *in vivo* dosimetry.
